# Use of paliperidone palmitate half-yearly release in patients diagnosed with psychotic disorder: profile and satisfaction of use

**DOI:** 10.1192/j.eurpsy.2023.1327

**Published:** 2023-07-19

**Authors:** P. Andres-Olivera, A. Alvarez, M. D. L. A. Garzon, R. Gonzalez, C. Roncero, P. Jesús

**Affiliations:** 1 Psychiatry, CAUSA; 2 Psychiatry, Salamanca University, Salamanca, Spain

## Abstract

**Introduction:**

The lack of insight can be present among patients with a diagnosis of schizophrenia, which often results in lack of adherence to pharmacological treatments^1^ and, subsequently, in treatment discontinuation and relapses^2^. This vicious pattern leads to further clinical deterioration, impaired functioning, and reduced quality of life ^3^. There is a plethora of evidence supporting the fact that long-acting injectable and depot antipsychotics can increase adherence to treatment, reduce the risk of discontinuation and hospital admissions^4^. It is also known that low fluctuations between peaks and lows in plasma drug levels could be related to a better tolerability profiles^5^. A new paliperidone palmitate prolonged release formulation, which is administered twice annually, has been approved as maintenance treatment for patients with schizophrenia who are already stable on the monthly or quarterly prolonged release paliperidone palmitate^6^.

**Objectives:**

We aimed to evaluate the transition of monthly and quarterly paliperidone palmitate to the new six-monthly formulation and patients’ satisfaction with it in a real-world clinical setting.

**Methods:**

We collected a basic epidemiologic questionnaire, responses to a query about local pain after administration, and the Drugs Attitude Inventory (DAI).

**Results:**

A total of 21 patients from an outpatient clinic for severe mental disorders with a long evolution of their disease in Salamanca, Spain, were included. All of them had a DSM5-TR diagnosis of Schizophrenia. Sixteen were male and 5 female. The mean age was 42.6 years. 14 were receiving quartlery paliperidone palmitate (10 with high doses (525 mg) and 4 with moderate doses (350 mg)) and 7 were on monthly injections (6 with high doses (150 mg) and 1 with a moderate dose (100 mg)). Those receiving moderate doses of quarterly or monthly paliperidone palmitate were administered 700 mg of six-monthly paliperidone palmitate; 1000 mg were injected to those with higher doses. The mean score on the DAI scale was 8. Only one patient reported an increase in local pain after the injection, and another reported dissatisfaction with the administration in the gluteus instead of the deltoid muscle. The first administration of the new formulation in our site was on June 26th; to date none of these patients have required hospital admission due to relapse.

**Conclusions:**

Six-monthly prolonged release paliperidone palmitate seems to be an effective maintenance treatment for schizophrenia. In addition, this new formulation is well received and tolerated by patients previously on monthly or quarterly formulations of the same drug.

**Disclosure of Interest:**

None Declared

